# Intra-articular osteoid osteoma of the talar neck initially managed as early avascular necrosis: a case report

**DOI:** 10.1093/jscr/rjag984

**Published:** 2026-10-27

**Authors:** Colton Evoniuk, Gabrielle Hartman, Erik Freeland

**Affiliations:** Department of Orthopaedics, Jacobs School of Medicine and Biomedical Sciences, 955 Main Street, Buffalo, NY 14203, United States; Department of Orthopaedics and Sports Medicine, University at Buffalo, 462 Grider Street, Buffalo, NY 14215, United States; Department of Orthopaedics and Sports Medicine, University at Buffalo, 462 Grider Street, Buffalo, NY 14215, United States

**Keywords:** osteoid osteoma, intra-articular lesion, talar neck, avascular necrosis, bone marrow edema

## Abstract

Intra-articular osteoid osteoma is an uncommon benign osteoblastic tumor that frequently presents with atypical clinical and radiographic findings, often delaying diagnosis. Lesions involving the talus are challenging because marrow edema and synovitis may obscure the characteristic nidus on magnetic resonance imaging (MRI), mimicking other pathologic processes. We present the case of a young male with a greater than 2-year history of atraumatic ankle pain and diffuse talar marrow edema on MRI, initially interpreted as osteonecrosis. He underwent ankle arthroscopy and talar core decompression, with minimal improvement. Repeat MRI demonstrated an evolving dorsolateral talar neck lesion, raising concern for osteoid osteoma and prompting excision. Histopathology demonstrated osteoid osteoma. At 6-month follow-up, he reported full pain resolution and unrestricted activity. This case highlights that intra-articular osteoid osteoma of the talus should be considered in young patients with persistent atraumatic ankle pain and diffuse marrow edema when the clinical course becomes discordant with avascular necrosis.

## Introduction

Osteoid osteoma is a benign osteoblastic neoplasm accounting for ~10% of benign bone tumors [1, 2]. Although most lesions arise within the cortex of the diaphysis of long bones, intra-articular lesions are less common and frequently present with atypical clinical manifestations [2]. The absence of classic nocturnal pain, variable response to nonsteroidal anti-inflammatory drugs (NSAIDs), and disproportionate synovitis may obscure the diagnosis [2].

The talus is an uncommon location for osteoid osteoma [3]. Because intra-articular lesions often generate extensive reactive bone marrow edema, magnetic resonance imaging (MRI) findings may be nonspecific and mimic stress injury, inflammatory arthropathy, osteochondral pathology, or osteonecrosis [2]. Consequently, diagnosis is frequently delayed [2].

We present a case of intra-articular osteoid osteoma of the talar neck initially managed as presumed early avascular necrosis based on serial MRI examinations and clinical findings. The diagnosis evolved only after persistent symptoms following initial operative treatment, prompting reassessment. To our knowledge, reports describing operative management for presumed talar osteonecrosis before eventual diagnosis of intra-articular osteoid osteoma are exceedingly uncommon.

## Case report

A 19-year-old healthy male presented with a greater than 2-year history of progressive atraumatic right ankle pain, denying any preceding injury or constitutional symptoms. Initial treatment consisted of activity modification, immobilization in a controlled ankle motion (CAM) boot, transition to a lace-up ankle brace, physical therapy, home exercises, and oral NSAIDs, all failing to provide sustained relief.

Physical examination consistently demonstrated preserved ankle and subtalar motion, full strength, and mild tenderness localized to the talus. No instability or neurovascular deficits were present.

Serial radiographs remained without evidence of talar collapse or significant degenerative change (Fig. 1). Initial MRI obtained in August 2023 demonstrated diffuse marrow edema throughout the talar dome, contributing to the initial diagnosis of early osteonecrosis (Fig. 2). Repeat MRI obtained in August 2024 demonstrated persistent but slightly decreased diffuse marrow edema throughout the talus with preservation of the talar dome (Fig. 3). Repeat MRI obtained in February 2025 again demonstrated persistent but slightly increased diffuse talar marrow edema, continuing to favor early osteonecrosis (Fig. 4).

**Figure 1:**
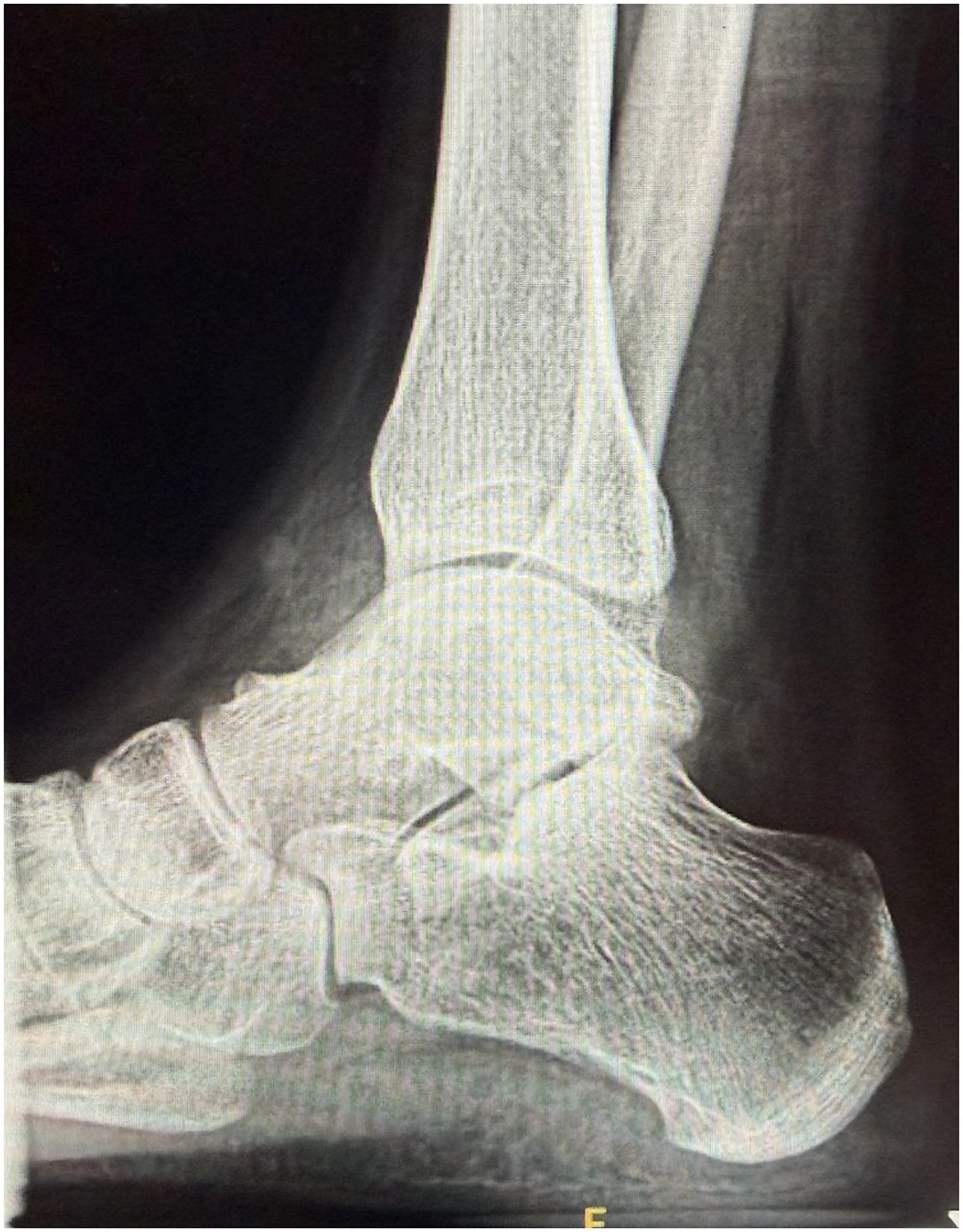
Lateral radiograph of the right ankle obtained during the initial evaluation demonstrates preserved tibiotalar alignment without evidence of talar dome or subchondral collapse.

**Figure 2:**
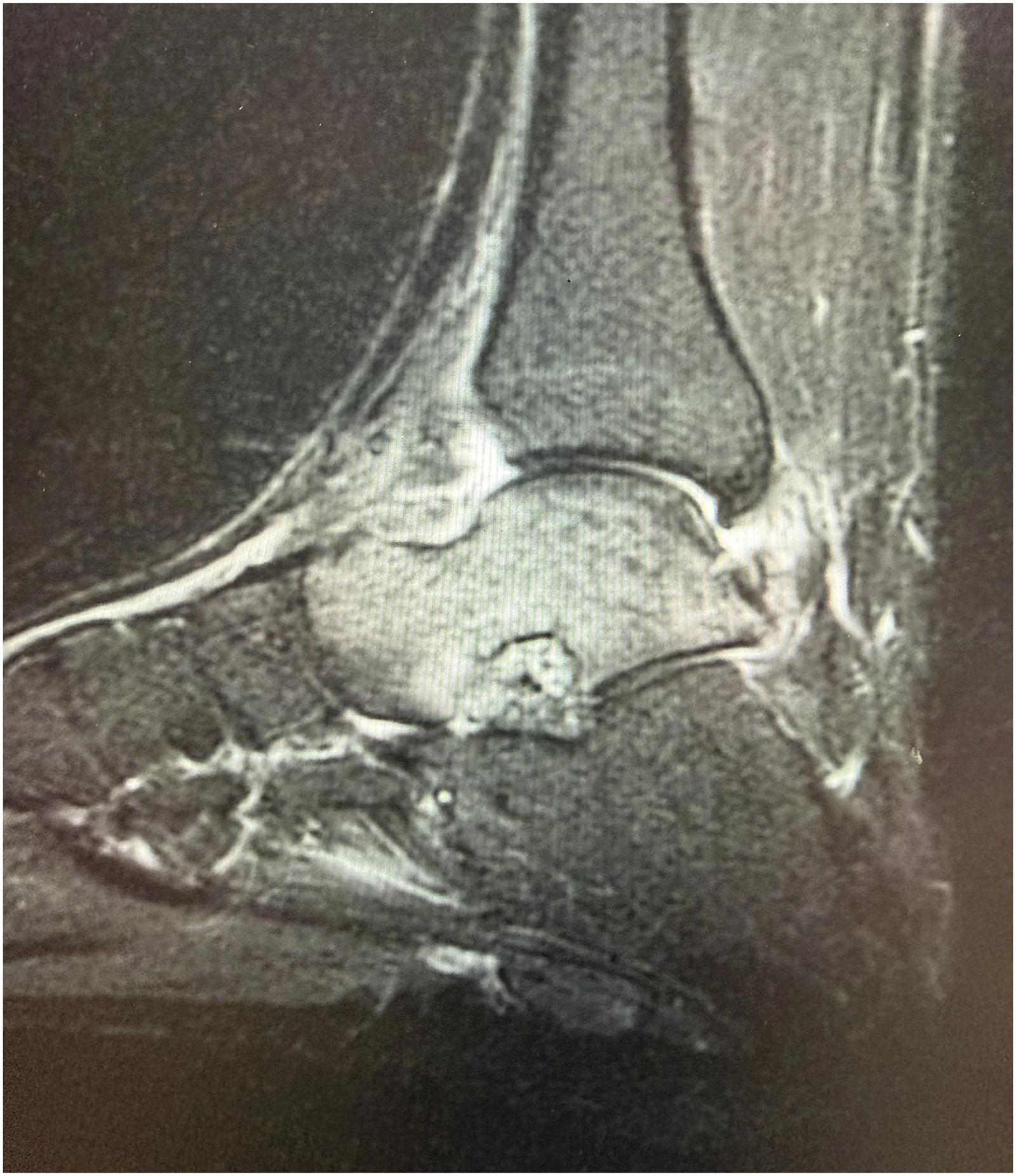
Sagittal T2-weighted fat-suppressed magnetic resonance image demonstrating extensive diffuse marrow edema throughout the talus with preservation of the talar dome. The marked reactive edema contributed to the initial diagnostic impression of early osteonecrosis.

**Figure 3:**
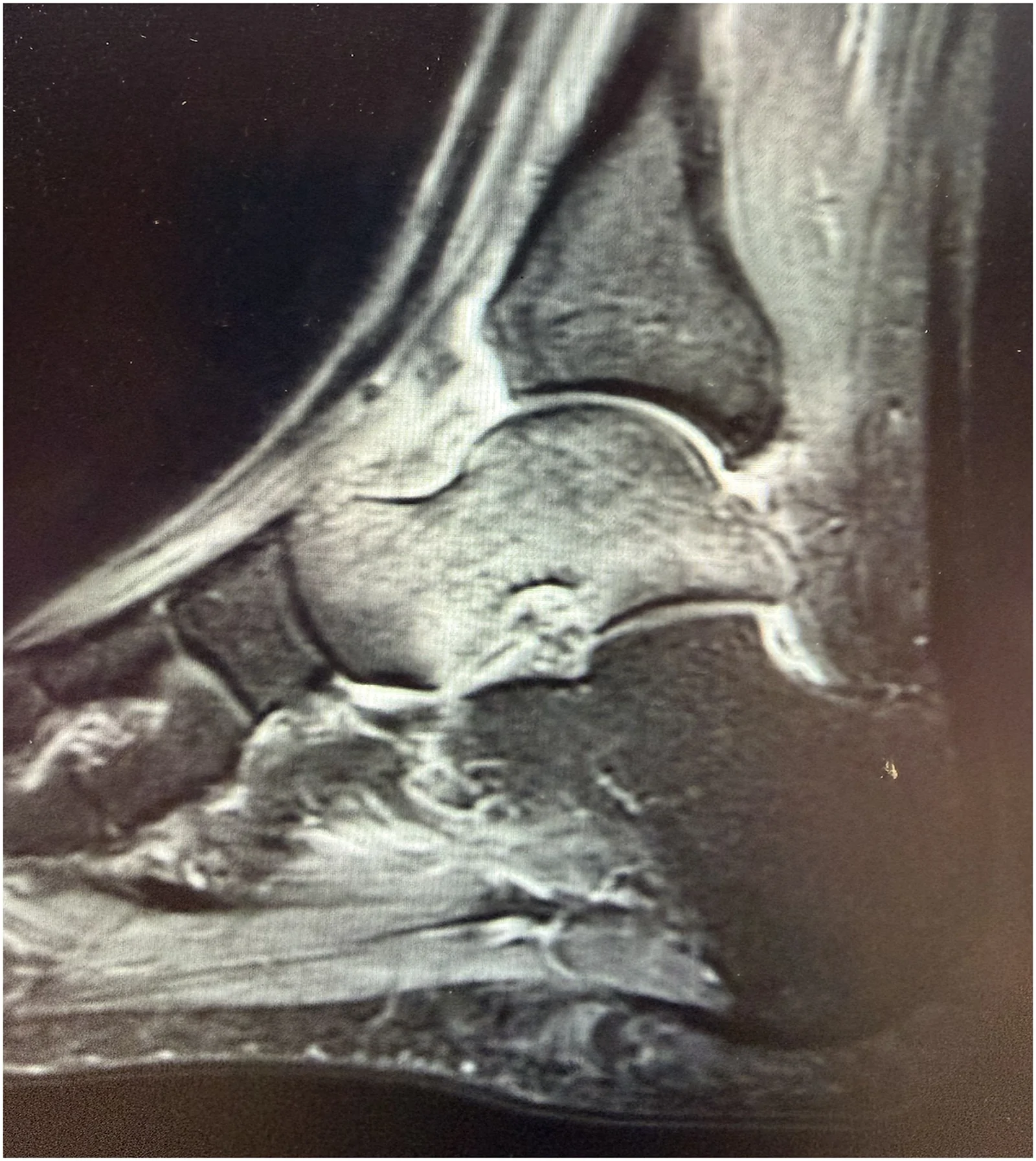
Sagittal T2-weighted fat-suppressed magnetic resonance image of the right ankle demonstrating persistent but decreased diffuse talar bone marrow edema compared with the prior MRI, with preservation of the talar dome articular surface.

**Figure 4:**
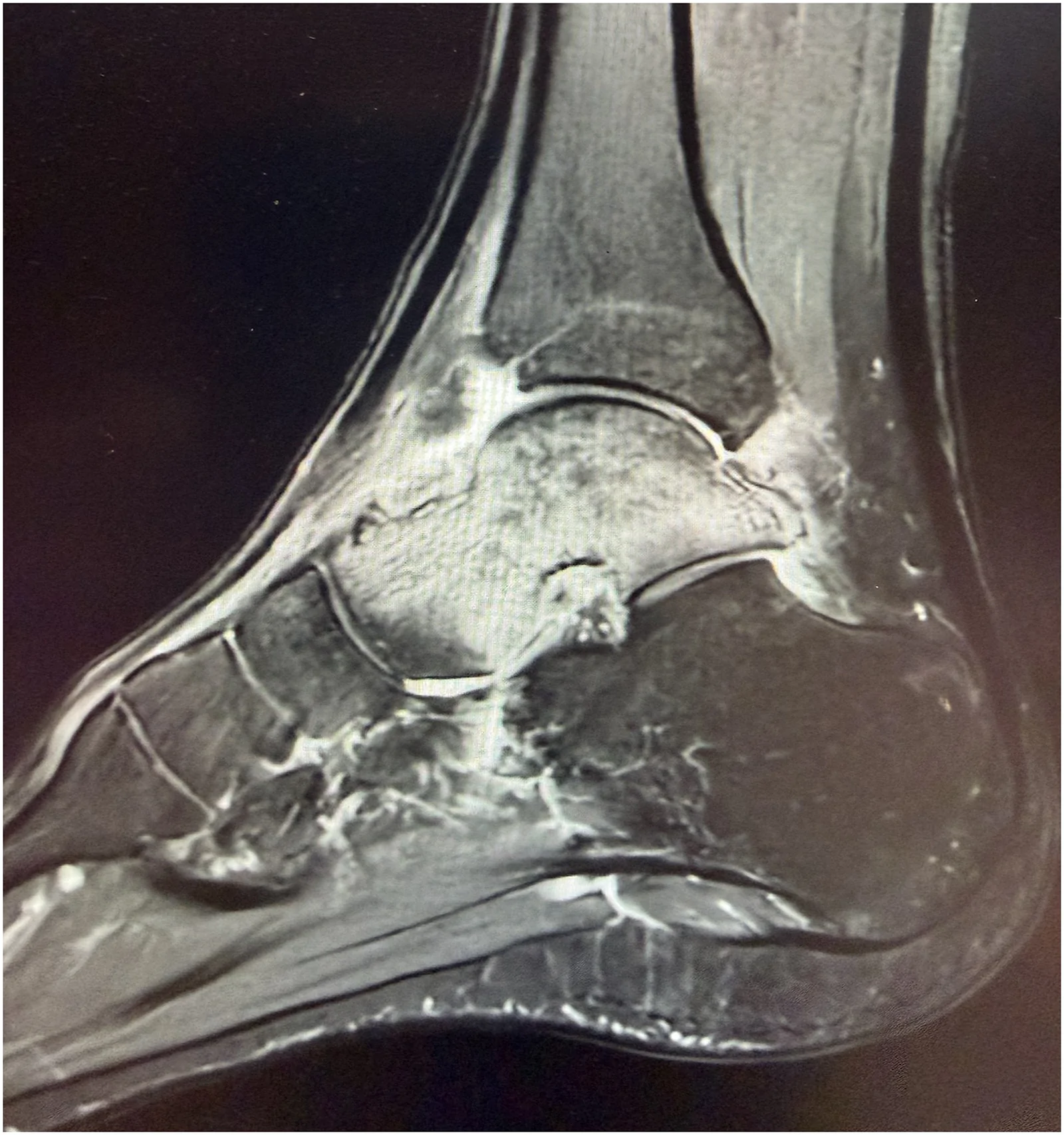
Sagittal T2-weighted fat-suppressed magnetic resonance image of the right ankle demonstrating persistent diffuse talar bone marrow edema with interval increase in marrow signal intensity compared to prior MRI. The talar dome remains preserved without evidence of subchondral collapse, supporting the working diagnosis of early osteonecrosis.

Given persistent symptoms despite prolonged conservative treatment, the patient elected for surgery. The patient underwent ankle arthroscopy with extensive synovectomy, core decompression of the talar body, and intraosseous bioplasty utilizing concentrated autologous bone marrow aspirate, demonstrating marked synovitis throughout the anterior ankle while the talar articular cartilage remained preserved (Fig. 5). A synovial biopsy was obtained.

**Figure 5:**
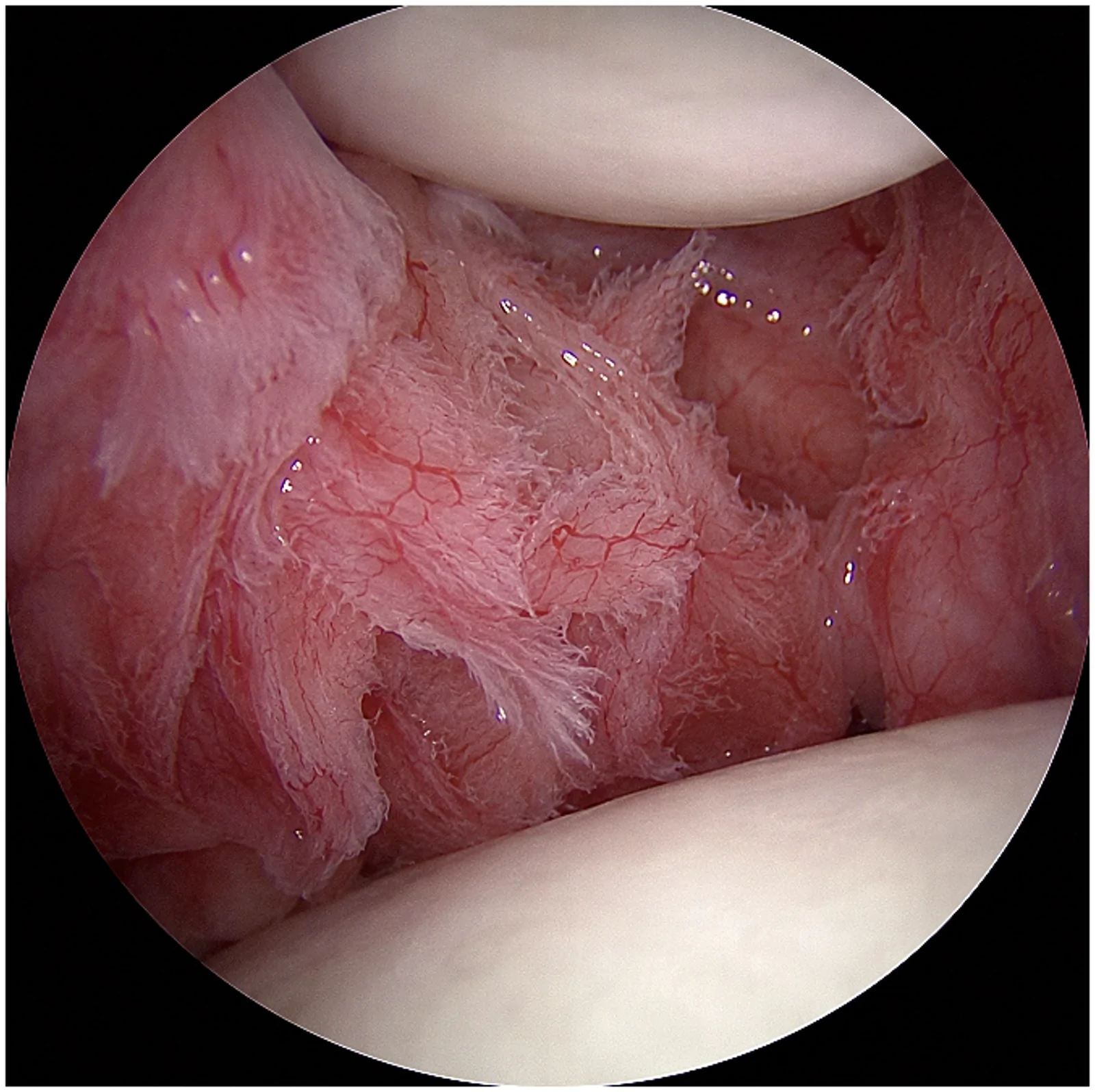
This image shows an arthroscopic view of the ankle with extensive, villous synovitis filling much of the anterior joint while the adjacent articular cartilage appears smooth and preserved.

Although the patient experienced mild symptomatic improvement, pain persisted. Follow-up radiographs along with updated MRI obtained 2 months following surgery demonstrated persistent diffuse talar marrow edema and a focal dorsolateral talar neck lesion with interval erosive change (Figs 6 and 7). Correlation of the updated imaging findings with the prolonged clinical course raised concern for intra-articular osteoid osteoma. Synovial biopsy demonstrated synovial lining hyperplasia and marked vascular proliferation. The patient continued to respond well only to oral NSAIDs, further suggesting osteoid osteoma.

**Figure 6:**
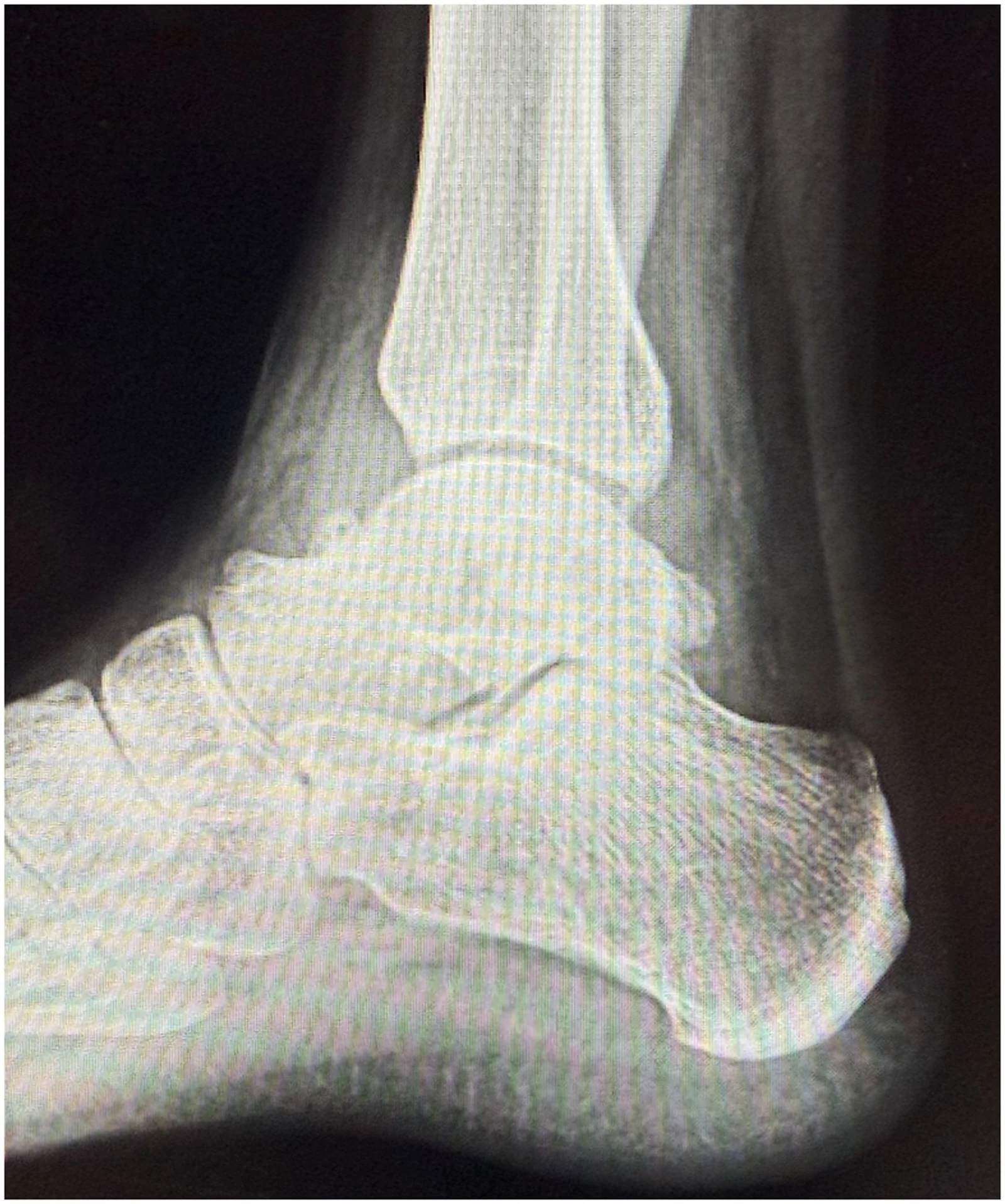
Lateral radiograph of the right ankle obtained 2 weeks following ankle arthroscopy, synovectomy, talar core decompression, and intraosseous bioplasty. Postoperative changes within the talar body are evident. Compared with the initial radiographs, a well-defined lucent lesion involving the dorsal aspect of the talar neck has become more conspicuous.

**Figure 7:**
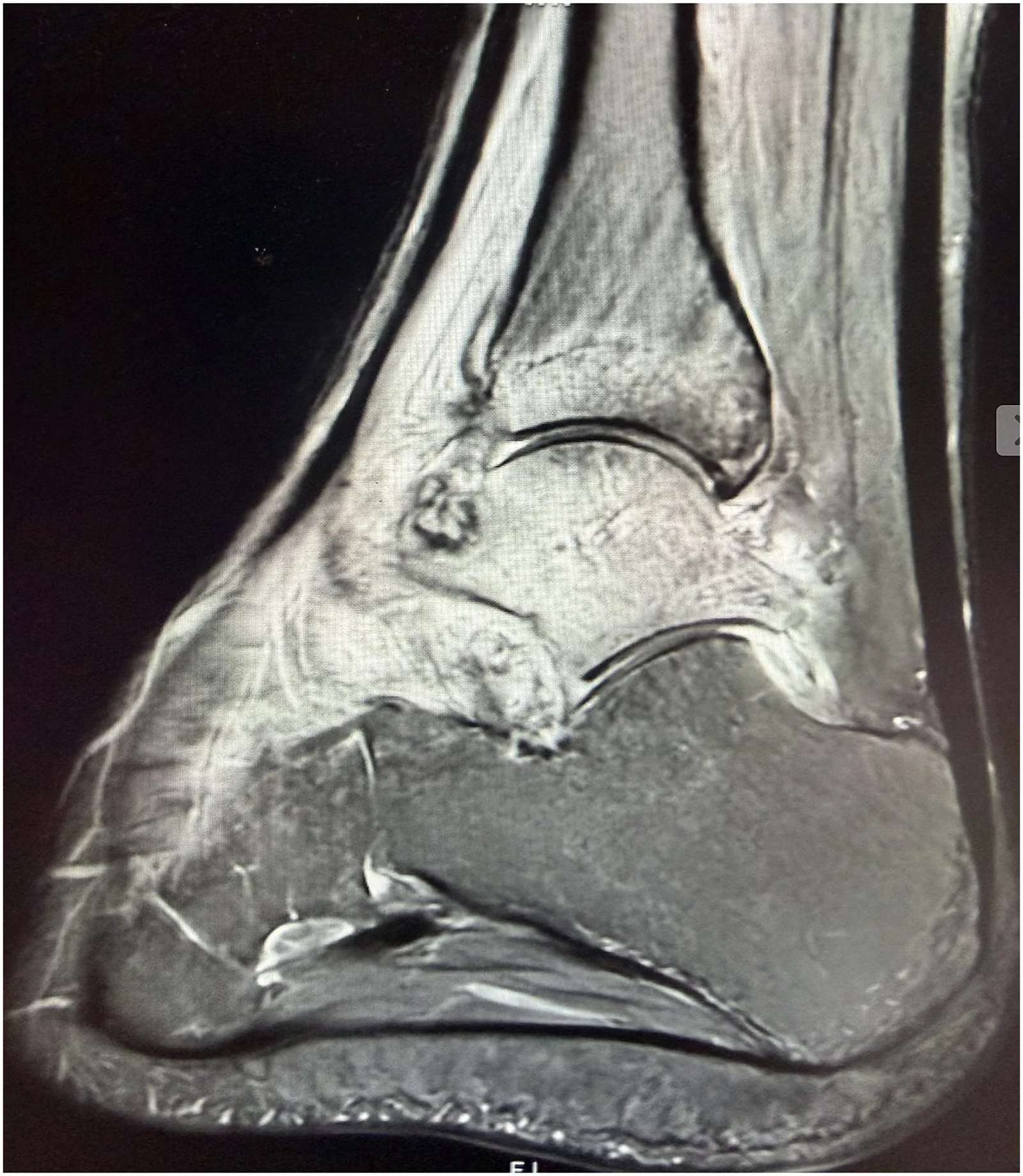
Sagittal T2-weighted fat-suppressed magnetic resonance image of the right ankle obtained ~2 months following ankle arthroscopy and talar core decompression. Persistent diffuse talar marrow edema is present with postoperative changes within the talar body. A well-defined focal lesion involving the dorsolateral talar neck with adjacent erosive change has become increasingly conspicuous compared with prior MRI examinations.

The patient subsequently underwent open excision and curettage of the dorsolateral talar neck lesion through an anterolateral approach (Fig. 8). The lesion measured ~1.5 × 1.5 cm and was excised under fluoroscopic guidance. The resulting defect was curetted thoroughly and filled with bone graft substitute. Histopathology demonstrated a benign osteoblastic lesion consistent with osteoid osteoma.

**Figure 8:**
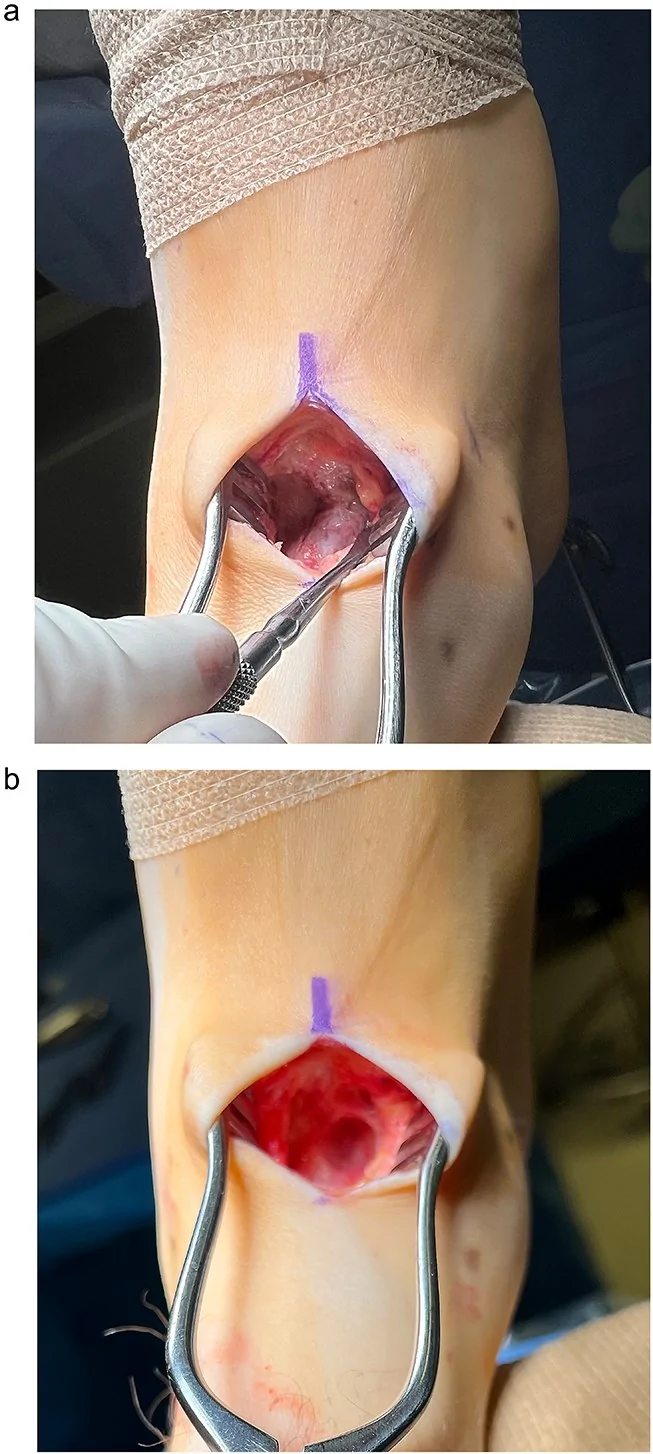
Intraoperative photographs demonstrating excision of the dorsolateral talar neck osteoid osteoma. (a) Exposure of the lesion following cortical window creation. (b) Osseous defect following complete curettage and excision, before bone grafting.

Postoperatively, the patient progressed uneventfully. He was initially immobilized in a short leg plaster splint before transitioning to a CAM boot at 2 weeks when he was permitted to bear weight to tolerance. By 8 weeks he had discontinued immobilization, resumed unrestricted weight-bearing activity, and reported marked improvement in pain. At 6-month follow-up, he had returned to full unrestricted activities, denied residual ankle pain, demonstrated full ankle motion and strength, and remained highly satisfied with his surgical outcome. Updated radiographs were obtained at that time (Fig. 9).

**Figure 9:**
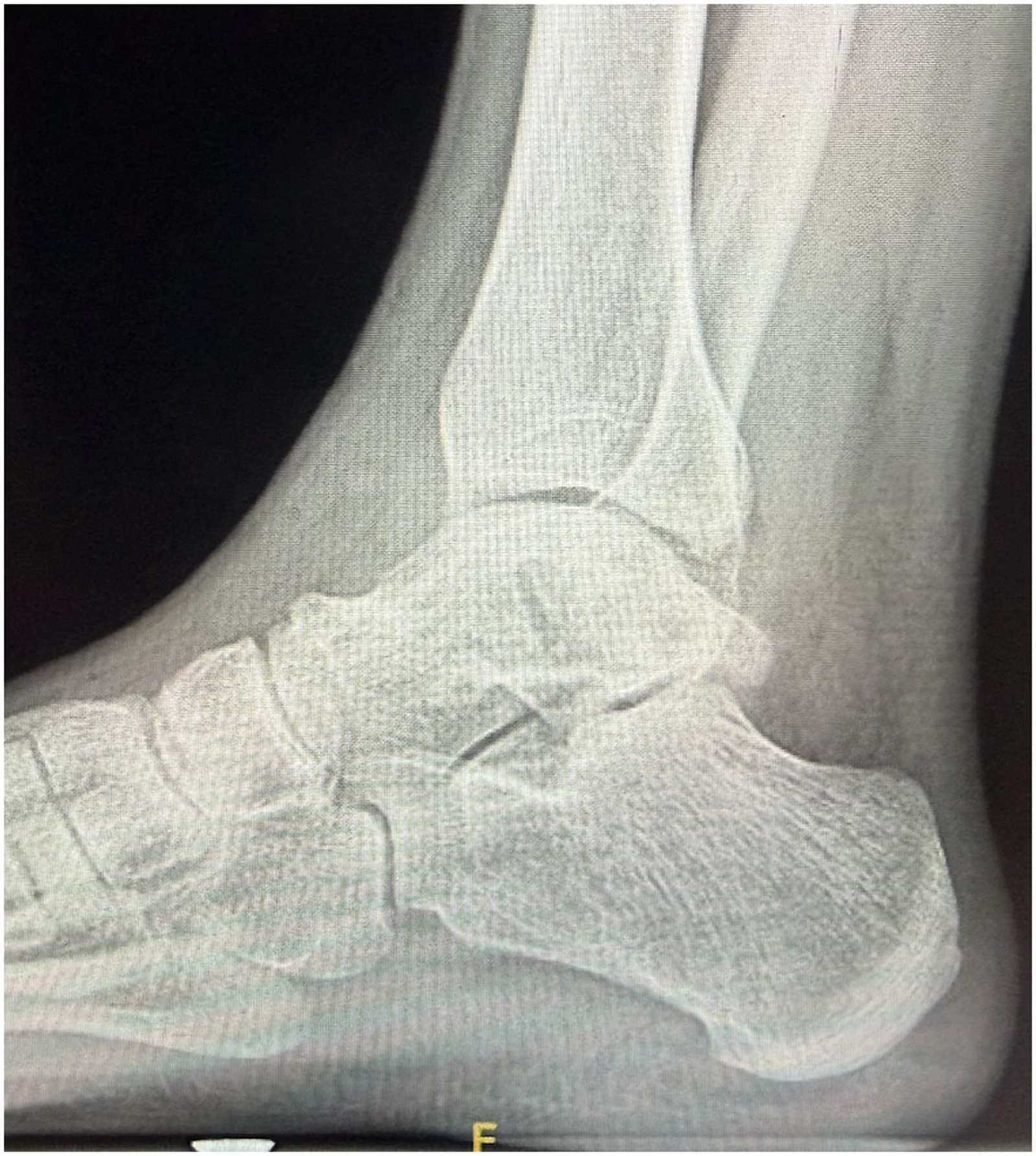
Final follow-up lateral radiograph obtained at 6 months after excision of the talar neck osteoid osteoma demonstrating maintained talar architecture, incorporation of the bone graft, and no evidence of recurrent lesion or talar collapse.

## Discussion

Intra-articular osteoid osteoma presents a diagnostic challenge because it frequently lacks the clinical and radiographic features of cortical osteoid osteoma [2, 3]. Rather than nocturnal pain with relief following NSAIDs, patients may present with chronic joint pain, synovitis, and marrow edema on MRI [2]. These atypical features often contribute to delayed diagnosis, highlighted by prior reports initially characterizing talar intra-articular osteoid osteoma as arthritis, tenosynovitis, or anterior impingement syndrome [4–6]. Similarly, this case highlights important diagnostic challenges associated with talar intra-articular osteoid osteoma.

MRI findings in patients with talar osteoid osteoma are often dominated by reactive inflammatory changes rather than direct nidus visualization [2]. A prior report identified MRI findings of edema, dorsal swelling, and subtalar effusion, leading to an initial misdiagnosis of synovitis [6]. Likewise, in our patient, serial MRI examinations consistently demonstrated talar marrow edema, joint effusion, and synovitis. Despite similar imaging presentations, the nonspecific pathologic features of osteoid osteoma pose significant diagnostic challenges. As a result, our findings reasonably supported stress injury and early osteonecrosis, but persistent symptoms and evolving imaging eventually prompted diagnostic reconsideration.

This case demonstrates the importance of correlating imaging with the overall clinical course. Despite prolonged symptoms, the patient lacked traditional risk factors and radiographic evidence of osteonecrosis. Arthroscopy demonstrated synovitis, but with preserved talar cartilage, findings that, in retrospect, favored an inflammatory intra-articular lesion over osteonecrosis. These unusual arthroscopic findings, along with persistent symptoms following core decompression, ultimately prompted reassessment. Arthroscopy has previously been performed prior to the diagnosis of osteoid osteoma; however, the misdiagnosis was synovitis [7]. Arthroscopic synovectomy newly identified an irregular tissue, with pathology confirming osteoid osteoma [7]. Although arthroscopy has been performed for presumed inflammatory conditions mimicking osteoid osteoma, our case is unique in that arthroscopic synovectomy was performed for presumed osteonecrosis. Altogether, our findings emphasize the importance of reconsidering diagnoses when persistent talar edema and the clinical course may suggest osteoid osteoma.

In addition, this case illustrates the complementary role of CT to evaluate persistent talar marrow edema. Although CT was not obtained in this case, thin-cut CT has been shown to improve identification of osteoid osteoma [2, 8]. With inconclusive MRI results, subsequent single-photon emission computed tomography (SPECT) with CT can raise suspicion for osteoid osteoma, with high-definition CT confirming the diagnosis [8]. When persistent talar marrow edema remains unexplained, integration of CT with the clinical course may facilitate earlier diagnostic reconsideration and help distinguish osteoid osteoma from mimics, preventing delays in treatment.
